# Supplementary material for: Multi-omics analysis of sarcospan overexpression in mdx skeletal muscle reveals compensatory remodeling of cytoskeleton-matrix interactions that promote mechanotransduction pathways
Source: Skelet Muscle. 2023 Jan 6;13:1. doi: 10.1186/s13395-022-00311-x (PMC9817407; doi:10.1186/s13395-022-00311-x)
Supplement: Supplementary file 1 — Additional file 1: Supplementary Figure 1. Enrichment analysis was performed using the Database for Annotation, Visualization and Integrated Discovery platform (DAVID, version 2021) and identified Gene ontology terms, denoted as nodes for both the RNA sequencing (right half of nodes) and mass spectrometry (MS) datasets (left half of nodes). Supplementary Figure 2. Gene expression from RNA sequencing of genes associated with YAP/TAZ signaling (a) or Wnt signaling (b) in WT, mdx, and mdxTG muscle in counts per million (CPM). Supplementary Figure 3. Additional images of indirect immunofluorescence analysis of 12-wk-old mouse quadriceps using an antibody against yes-associated protein 1 (Yap1) showing increased Yap1 signal in mdx and mdxTG tissue showing possible immune cell Yap1 staining in mdx tissue. Supplementary Table 1. Summary of SSPN-Tg murine lines. Supplementary Table 2. RNA_ECM. Supplementary Table 3. RNA_Actin Cytoskeleton. Supplementary Table 4. Ingenuity Pathway Analysis – RNAsequencing WT vs mdx. Supplementary Table 5. Ingenuity Pathway Analysis – RNAsequencing WT vs mdxTG. Supplementary Table 6. Ingenuity Pathway Analysis – RNAsequencing mdx vs mdxTG. [file 13395_2022_311_MOESM1_ESM.zip › McCourt Stearns-Reider et al_Supplemental Material_second revision_final.pdf]

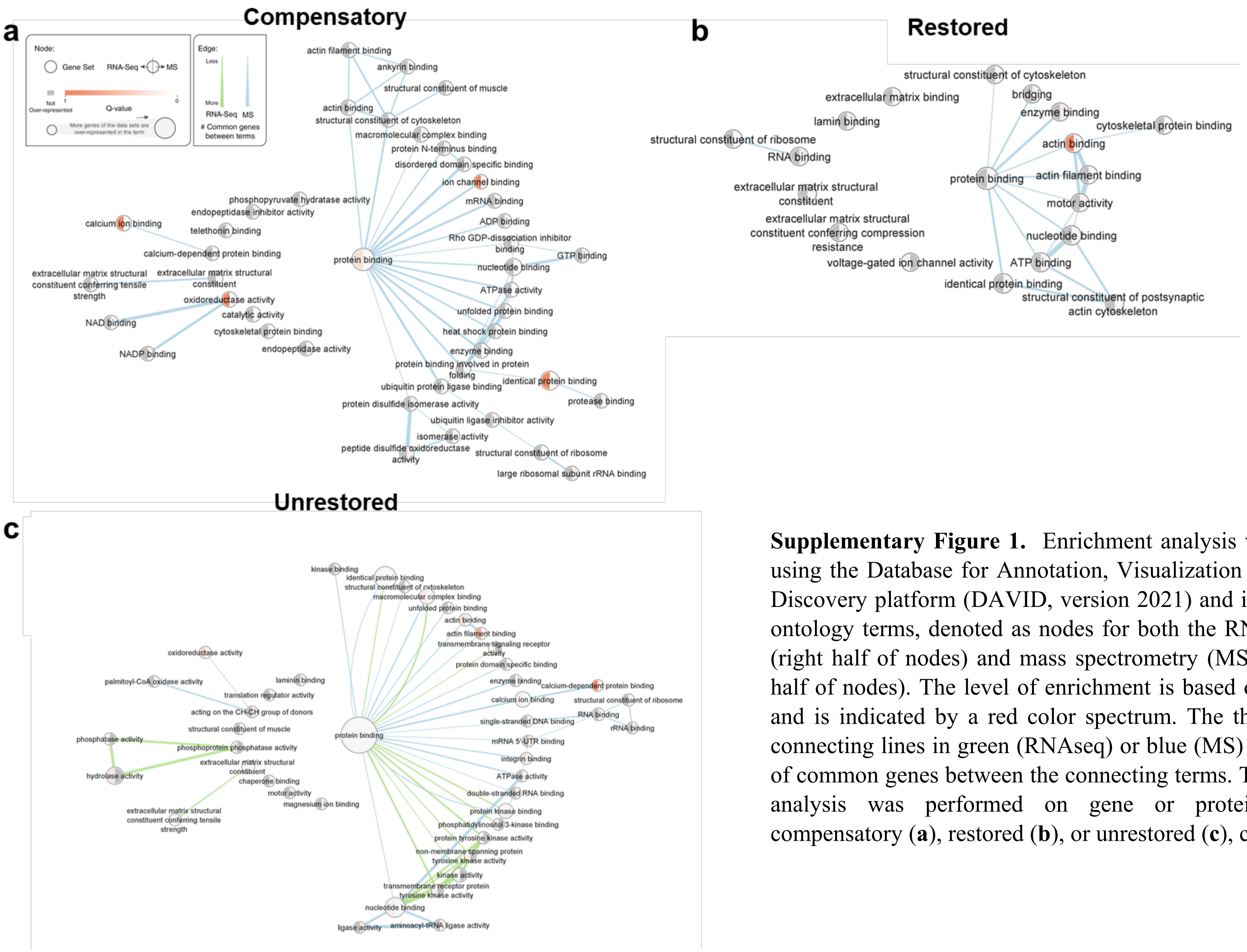

Supplementary Figure 1. Enrichment analysis was performed using the Database for Annotation, Visualization and Integrated Discovery platform (DAVID, version 2021) and identified Gene ontology terms, denoted as nodes for both the RNA sequencing (right half of nodes) and mass spectrometry (MS) datasets (left half of nodes). The level of enrichment is based on the Q-value and is indicated by a red color spectrum. The thickness of the connecting lines in green (RNAseq) or blue (MS) indicates the # of common genes between the connecting terms. The enrichment analysis was performed on gene or protein lists from compensatory (**a**), restored (**b**), or unrestored (**c**), categories.

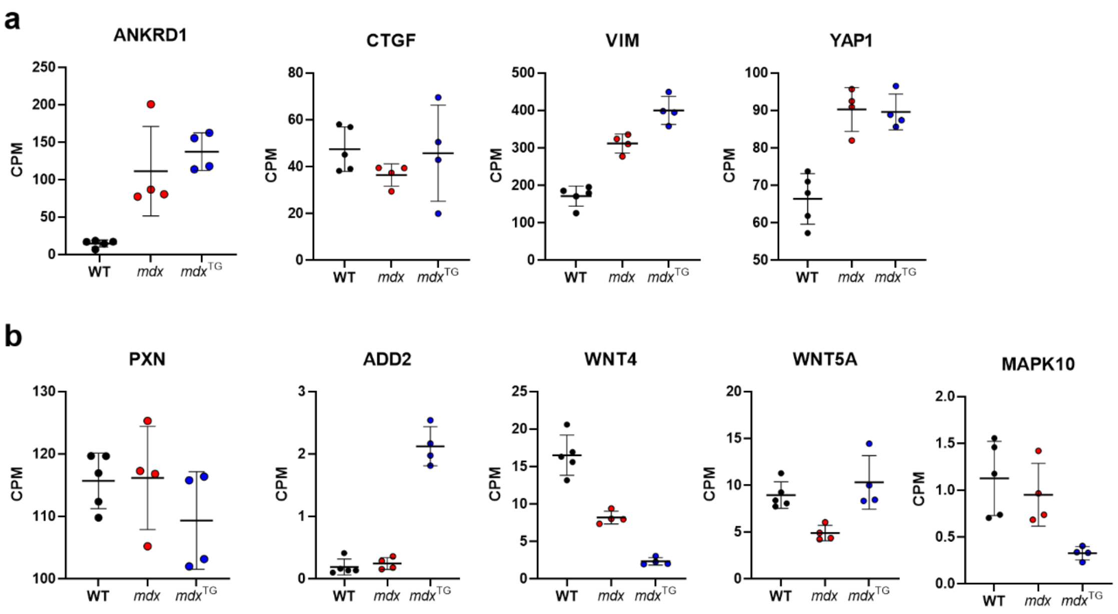

Supplementary Figure 2. Gene expression from RNA sequencing of genes associated with YAP/TAZ signaling (a) or Wnt signaling (b) in WT, *mdx*, and *mdx* ^{TG} muscle in counts per million (CPM). ANKRD1: Ankyrin Repeat Domain 1; CTGF: connective tissue growth factor; VIM: vimentin; YAP1: yes associated protein 1; PXN: paxillin; ADD2: adducin 2; WNT4: wingless-type MMTV integration site family, member 4; WNT5A: wingless-type MMTV integration site family, member 5a; MAPK10: mitogen-activated protein kinase 10.

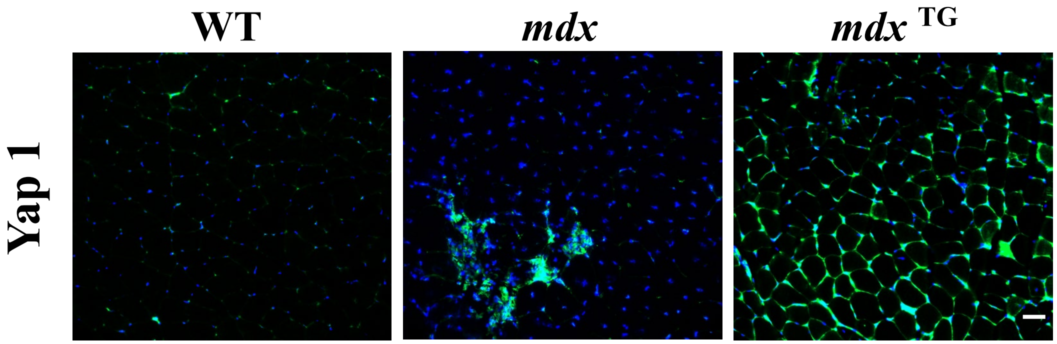

Supplementary Figure 3. Additional images of indirect immunofluorescence analysis of 12-wk-old mouse quadriceps using an antibody against yes-associated protein 1 (Yap1) showing increased Yap1 signal in *mdx* and *mdx* ^{TG} tissue showing possible immune cell Yap1 staining in *mdx* tissue. More representative images are reported in Figure 7. Scale bar = 100 μm.

Supplementary Table 1. Summary of SSPN-Tg murine lines

	Effect of SSPN transgene on C57Bl/6J phenotype			*Effect of SSPN transgene on mdx phenotype*				
**SSPN-Tg line**	**Tg***	**SSPN level (fold)**	**Phenotype**	**Central nucleation**	**Membrane damage**	**Adhesion complexes at the sarcolemma**	**SkM fibrotic scarring**	**Cardiac phenotype**
28	M	30	Healthy[30]	Reduced pathological regeneration[30]	Improved membrane integrity[30-31]	Increased UGC[30], ITGB[30-31]	Reduced fibrotic scarring[30]	Improved systolic and diastolic function[31]; reduced fibrosis[31]
3	H	3	Healthy[35]	Reduced pathological regeneration[35-36]	Improved membrane integrity[29,35]	Increased UGC[29.35], ITGB[35**], SPTBN1[**]	Reduced fibrotic scarring[47]	Improved systolic and diastolic function[29]; reduced fibrosis[29]
29.1	H	2-3	Healthy[32]	Reduced pathological regeneration[27]	Improved membrane integrity[27]	Increased UGC[27]	n.d.	n.d.
31.6	H	2-3	Healthy[32]	Data similar to line 29.1 (unpublished).				
36.6	H	2-3	Healthy[32]	Line not created on *mdx* background due similarity with lines 29.1 and 31.6.				
31.7	H	10	Premature lethal due to protein aggregation[32]	Line not created on *mdx* background due to early lethality in C57Bl/6J.				
37.5	H	10	Premature lethal due to protein aggregation[32]	Line not created on *mdx* background due to early lethality in C57Bl/6J.				
0.5	H	0.5	Healthy[35]	No effect[35]	n.d.	No effect[35]	n.d.	n.d.
1.5	H	1.5	Healthy[35]	No effect[35]	n.d.	Increased UGC[35], ITGB[35]	n.d.	n.d.

*Species origin of SSPN transgene (H: human; M: mouse). Gray shading indicates murine lines used in this manuscript. **Data from this manuscript. UGC: utrophin glycoprotein complex; ITGB: integrin complex; SkM: skeletal muscle; Tg: transgenic; n.d.: not determined.

Supplementary Table 4. Ingenuity Pathway Analysis – RNAsequencing WT vs *mdx*

*Target genes in red are ECM, ECM-associated, cytoskeletal, or cytoskeletal-associated genes.

Predicted Activation State	Upstream Regulator	Molecule Type	Target Genes	Activation z-score	p-value of overlap
**Inhibited**	let-7	microRNA	AURKB, BCAT1, BRCA1, BUB1, BUB1B, CASP3, CCNA2, CCNB1, CCNF, CD44, CDC20, CDCA2, CDCA3, CDCA7, CDCA8, CDK1, CDK6, CDT1, **COL3A1**, **DMD**, E2F8, FANCD2, FOSL1, ID2, IGF2BP2, MCM5, NUF2, PLAGL1, PLAUR, RRM2, SKP2, SOX9	-4.845	1.42E-10
	Alpha catenin	actin assembly	**ADAM8**, **ADAMTS4**, BCL3, C1QTNF3, **COL15A1**, **COL3A1**, **COL6A3**, EPHA3, IGF2 , IL2RG, **ITGAM**, LUM, LYZ, **MMP12**, **MMP19**, SOCS3, **TIMP1**, TNFAIP2, TNFRSF12A, VCAM1	-4.406	1.85E-07
	mir-21	microRNA	AIF1, ANLN, ASPM, BCL2, CCNA2, CCNB1, CD180, CDK6, CDKN1A, CDKN2A, CLEC5A, COL3A1, CSF3R, DDAH1, DLGAP5, ECT2, FCGR1A, FCGR2A, FGL2, IFI16, IGHM, INPP5D, IRGM, KIF23, KIF4A, KIFC1, KNTC1, MKI67, NPAS2, NUSAP1, OAS3, PBK, PER2, PRC1, RAB7B, RACGAP1, RAD51AP1, Slfn2, SMC2, STMN1, **TGFB1**, TIAM1, TLR1, TOP2A, TRAF1, TREM2, VCAM1, ZWILCH	-4.166	9.62E-17
	Apolipoprotein E (APOE)	transporter	ADGRE1, APOE, ATF3, C5AR2, CASP1, CASP3, Ccl2, CCL22, CCR5, CD44, CD68, CTSS, CYBB, DAGLA, EGR1, FCGR1A, FCGR2B, FOS, IL10RA, IL1RN, **ITGAM**, **ITGAX**, LIPA, LPL, LRP8, **MMP3**, MSR1, NCF1, NCF2, NPNT, PRKCB, SERPINA3, SOCS3, SPP1, TCIRG1, TGFB1, TIAM1, TIFA, **TIMP1**, TNIP1, TREM2, UCP2, VCAM1	-3.966	4.89E-11
	immunity-related GTPase family M member 1 (IRGM1)	GTPase	AURKB, BUB1, Ccl2, CCNA2, CCNB1, CCNB2, CDCA3, DTL, ID2, IFI16, KIF20A, LILRB4, MKI67, NCAPG, NEK2, RRM2	-3.93	1.45E-10
**Activated**	Vascular endothelial growth factor (VEGF)	growth factor	ALOX5AP, ANPEP, ARHGAP22, ASNS, ATF3, AURKB, BCL2, BIRC5, BUB1, BUB1B, CASP1, CASP3, Ccl2, CCNF, CD44, CDC20, CDK1, CDKN2A, CENPF, CLDN1, CNTFR, CXCR4, DUSP5, EGR1, EMP1, FOSL1, FOXM1, GJB2, GPRC5B, GPSM2, GRB10, HELLS, HPSE, KIF11, KIF15, KIF20B, KIF22, LRP8, LY75, MCM5, MELK, MKI67, **MMP14**, Mt2, NEK2, NRCAM,	5.457	3.46E-13

Predicted Activation State	Upstream Regulator	Molecule Type	Target Genes	Activation z-score	p-value of overlap
			PLAUR, **PLK1**, **PLK4**, PRC1, PRKCB, SCML4, SFN, SKP2, SMC2, SOCS3, TGFB1, TIMP1, TNFRSF11A, VAV3, VCAM1		
	Forkhead Box O1 (FOXO1)	transcription regulator	ANLN, ASPM, ATP6V0D2, BCL2, BID, BIRC5, C1QA, CCNA2, CCNB1, CCNB2, CCNF, CDK1, CDKN1A, CDKN2A, CENPF, CIDEA, DCX, DIO2, DLGAP5, EGR1, FABP5, FOS, FOXM1, IKZF1, IL17RA IL7R, **ITGAM**, **ITGB2**, KIF11, LCP2, LPL, MCM5, **MMP3**, **MYL3**, MYOG, NCAPG, NEK2, NUSAP1, PAK1, PBK, PRC1, PTPRC, Ptprv, RUNX2, SFN, SLC25A22, SLC25A24, SPC25, SQLE, **TGFB1**, TNFRSF11A, TRAF1, TSPO, VCAM1, WNT4	5.554	7E-13
	Interferon Gamma (IFNG)	cytokine	ADCY7, ADRA2A, AIF1, AIF1L, ALOX5AP, ASNS, ATF3, BCL2, BCL3, BID, BMP6, BST1, C1QA, C1QB, C1QC, CASP1, CASP3, Ccl2, CCL22, CCL28, Ccl7, Ccl8, CCNA2, CCR1, CCR5, CD14, CD276, CD4, CD44, CD55, CD68, CD72, CD83, CDK5R1, CDKN1A, CDKN2A, CELSR2, CERS6, CHRND, CHRNG, CIITA, CLEC5A, CLEC7A, CORO1A , CSF2RB, **CTSH**, **CTSS**, CX3CR1, CXCL16, CXCR4, CYBA, CYBB, DUSP5, E2F1, EFCAB6, EGR1, FABP5, FCER1G, FCGR1A, FCGR2A, FCGR2B., FCGR3A/FCGR3B, FGL2, FOS, GDF15, GJB2, GPRC5B, HCK, HLA-DMB, IFI16, IFI30, IKBKE, IL10RA, IL17RA, IL18RAP, IL1RN, IL7R, IRF8, IRGM, **ITGAL**, **ITGAM**, **ITGAX**, **ITGB2**, CNMA1, KLF10, **LAMC2**, LAT2, LCP2, LGALS3, LPL, MEFV, MERTK, **MMP12**, **MMP3**, Ms4a4b (includes others), MSR1, Mt1, MYOG, NAPSA, NCAM1, NCF2, NEURL3, OAS3, P2RY6, PARVG, PBK, PLA2G5, PLAUR, PLEK, PTAFR, PTPN6, RAC2, RUNX2, RUNX3, SBNO2, SGPL1, SLC11A1, SLC15A3, SOAT1, SOCS3, SPI1, **SPP1**, SPRR1A, SQLE, STX11, TBXAS1, TCIRG1, TFRC, **TGFB1**, THEMIS2, **TIMP1**, TLR1, TLR6, TLR7, TLR8, TNFAIP2, TNFRSF11A, TNFRSF12A, TNFRSF1B, TREM2, TYROBP, VCAM1, Wfdc17	6.595	8.6E-24
	Tumor necrosis factor (TNF)	cytokine	AATK, ABCC3, **ADAM8**, **ADAMTS4**, AKR1B10, ALCAM, ALOX5AP, ANPEP, APOE, ARHGAP22, ATF3, B4GALNT1, BCL2, BCL3, BID, BIRC5, BUB1B, C3AR1, CASP1, CASP3, Ccl2, CCL22, CCL28, Ccl7, CCNE1, CCR1, CCR5, CD14, CD247, CD4, CD44, CD55,	7.103	8.45E-24

Predicted Activation State	Upstream Regulator	Molecule Type	Target Genes	Activation z-score	p-value of overlap
			CD83, CDK5R1, CDKN1A, CDKN2A, CERS6, CHRND, CHRNG, CIITA, CLEC5A, CMBL, CNR2, **COL15A1**, **COL3A1**, COTL1, CSF2RB, **CTSS**, CX3CR1, CXCL16, CXCR4, CYBA, CYBB, CYP1B1, CYTIP, **DMD**, DUSP14, DUSP5, E2F1, EFHD2, EGR1, ELF3, EMP1, EXOC3L4, FABP5, FCER1G, FCGR2B, FOS, FOSL1, FRZB, FUT4, GABRA1, GDF15, GPR176, GPRC5B, GRN, H19, HEXB, HGF, HK3, Hmgn2 (includes others), HPGDS, IDH2, IFI16, IGF2, IKBKE, IL10RA, IL1RN, IL21R, IL7R, INPP5D, IRF8, **ITGAL**, **ITGAM**, **ITGAX**, **ITGB2**, KCNH2, KIF20A, KLF10, KLF5, KRT18, **LAMC2**, **LGALS3**, LPL, MBP, MEFV, **MMP12**, **MMP14**, **MMP3**, MSLN, MSR1, MSTN, Mt1, Mt2, MYOG, NAIP, NCAM1, NCF1, NCF2, OAS3, P2RY6, PCDH7, PDK3, PDPN, PER2, PLA2G5, PLAUR, PLD3, **POSTN**, PTPRC, PYCARD, RAB32, RASSF7, RGS1, RGS16, RRAD, RRM2, RUNX2, SCUBE2, SELPLG, SERPINA3, SERPINB1, SGPL1, SLC11A1, SLC15A3, SLC22A4, Slfn2, SOAT1, SOCS3, SOX9, **SPP1**, SQLE, STMN1, TBXAS1, TFRC, **TGFB1**, TIFA, **TIMP1**, TLR7, TLR8, TNFAIP2, TNFRSF11A, TNFRSF1B, TNIP1, TRAF1, TREM2, UCP2, VAV3, VCAM1, WISP1, ZNF365, ZNF750		
	Colony stimulating factor 2 (CSF2)	cytokine	ABCG1, ADAl, **ADAM8**, ALOX5AP, ANLN, BCL2, BCL3, BID, BIRC5, BUB1, BUB1B, CASP1, CASP3, Ccl2, Ccl8, CCNA2, CCNB1, CCNF, CCR1, CCR5 CD14, CD180, CD276, CD83, CDC20. CDCA2, CDCA3, CDCA8, CDK1, CDKN1A, CENPE, CIITA, CLEC6A, CLEC7A, **COL8A1**, CSF2RA, CSF2RB, CXCR4, CYBB, E2F8, EGR1, FANCA, FCGR1A, FCGR2B, FIGNL1, FOS, FOSL1, FOXM1, GDF15, HGF, ID2, IL1RN, INPP5D, **ITGAM**, **ITGAX**, KIF11, KNTC1, LCP1, LY75, LY9, MCM5, MKI67, **MMP14**, MNS1, NEK2, NUSAP1, PLK1, PRC1, PTGER2, PTK2B, RACGAP1, **RHOH**, RRM2, SLAMF7, Slfn2, SMC2, SNTB1, SOCS3, SPC25, SPI1, **SPP1**, STMN1, **TGFB1**, TIFA, TLR1, TNFRSF11A, TNFRSF1B, TOP2A, UHRF1	7.612	4.74E-32

Supplementary Table 5. Ingenuity Pathway Analysis – RNAsequencing WT vs *mdx* TG

*Target genes in red are ECM, ECM-associated, cytoskeletal, or cytoskeletal-associated genes.

Predicted Activation State	Upstream Regulator	Molecule Type	Target Genes	Activation z-score	p-value of overlap
**Inhibited**	Alpha catenin	actin assembly	**ADAM8**, **ADAMTS12**, **ADAMTS4**, BCL3, BGN BIRC3, CDH11, **COL15A1**, **COL3A1**, **COL5A1**, **COL5A2**, **COL6A1**, **COL6A2**, **COL6A3**, **CTSB**, CXCL10, EPHA3, IGFBP4, **ITGA5**, KLK3, **LUM**, LYZ, **MMP12**, **MMP19**, **MMP2**, PTGS2, RELB, **RHOC**, **TIMP1**, **TNC**, TNFAIP3, TNFRSF12A, **VIM**	-5.581	8.36E-12
	let-7a-5p (and other miRNAs w/seed GAGGUAG)	mature microRNA	AURKB, BIRC5, CAPG, CASP3, CCNE1, CDK6, CDKN2A, **COL27A1**, **COL3A1**, DOCK5, FADS2, HMGA1, Hmga2, IGF2BP2, PTGS2, **RHOG**, S100A4, SLC25A24, SMOX, THBS1, TLR4, UHRF1, **VIM**	-4.24	0.00064
	miR-124-3p (and other miRNAs w/seed AAGGCAC)	mature microRNA	ANXA8/ANXA8L1, CDK6, CHSY1, EGR1, ELOVL1, FAM129B, FAM83H, GAS2L1, HTATIP2, INO80C, KLF15, LDLR, LITAF, MDFIC, NME4, OAF, PGF, PGM1, PLP2, RASSF5, RBM47, **RHOG**, SERPINB6, SOX9, STOM, SUCLG2, TMBIM1, TSC22D4, **TUBB6**, UHRF1, ZFP36L2	-4.172	0.000432
	Apolipoprotein E (APOE)	transporter	ACAT1, ADGRE1, ALOX12, ATF3, BEGAIN, BGN, CASP3, CCL22, CCL5, CCR5, CD44, CD68, CD80, CD86, CLU, **COL18A1**, CREB3L1, **CTSB**, **CTSK**, **CTSS**, Cyb5r3, CYBB, EGR1, EMILIN1, F2R, F2RL1, FOS, GLUL, GRIA3, HSPA1A/HSPA1B, Hspa1b, HSPA5, IL10RA, IL12A, IL1RN, **ITGA5**, **ITGAX**, KCNAB1, LDLR, LPL, LRP8, **MMP2**, **MMP3**, NCF1, PC, PHLDA1, PRKCB, PTGS2, PTP4A3, SERPINA3, SREBF2, **TGFB1**, TIAM1, **TIMP1**, TNIP1, TREM2, UCP2	-3.727	1.88E-10
	Estrogen receptor	group	ANXA1, BCL2, CAPG, CD44, CD68, CDH11, CDH19, CDH4, CNKSR1, **COL4A1**, **COL4A2**, **COL4A5**, **COL5A1**, **COL6A1**, **COL6A2**, EGFR, EGR1, ERBB2, **FLNC**, FOS, GSTA5, HBEGF, HSPA1A/HSPA1B, KLF10, KRT18, KRT7, KRT8, **LAMC2**, LDLR, **LOXL2**, MAP1B, **MMP14**, MSN, PCDH7, PLAUR, RGS2, **TGFB1**, **TIMP1**, TLR4, TNC, **VIM**	-3.661	1.34E-07
**Activated**	Platelet-derived growth factor subunit BB	complex	ATF3, BCL3, BMP6, BRCA1, CASP4, CCNE1, CD44, CDK1, CDKN1A, **COL18A1**, **COL3A1**, CRYAB, CTH, DUSP5, EGFR, EGR1, EGR2, EGR3, FASN, FHL1, FOS,	6.167	2.46E-17

Predicted Activation State	Upstream Regulator	Molecule Type	Target Genes	Activation z-score	p-value of overlap
	(PDGF BB)		FOSB, FOSL1, FZD1, GADD45A, GDF15, GEM, GLUL, GSS, H19, HBEGF, HLA-E, Hmga2, IER2, IGFBP4, **ITGA5**, JUNB, KLF10, LDLR, **LGALS3**, **LMNA**, **MMP12**, **MMP14**, **MMP2**, **MMP3**, Mt1, Mt2, PHLDA1, PLAT, PLK2, **POSTN**, PRRX2, PTGS2, Pvr, RGS1, RGS2, RXRG, S1PR2, Scd2, SERPINA3, SLC2A3, SLC7A1, SPHK1, TEAD4, **TGFB1**, THBS1, **TIMP1**, TNC, TNFAIP3, TNFRSF12A, VCAN		
	Colony stimulating factor 2 (CSF2)	cytokine	ADA, **ADAM8**, ADGRE5, **ANXA1**, ATXN1, AURKA, BBC3, BCL2, BCL3, BID, BIRC3, BIRC5, BUB1, BUB1B, CASP3, CCNA2, CCNB1, CCR5, CD14, CD180, CD276, CD63, CD80, CD83, CD86, CDCA2, CDCA8, CDK1, CDKN1A, CENPE, CIITA, CKS1B, CLEC6A, CLEC7A, **COL8A1**, CXCL10, CYBB, E2F8, EGR1, EGR2, EGR3, F2R, F2RL1, FIGNL1, FOS, FOSL1, FOXM1, GDF15, HBEGF, HGF, IL1RN, **ITGAX**, JAK2, JUNB, KIF11, LCP1, LY9, LY96, MKI67, **MMP14**, **MMP2**, NFKB2, PRC1, PTGER2, PTGS2, PTK2B, QSOX1, REC8, RELB, RRM2, SLAMF7, SLC2A1, SLC2A3, SLC2A4, SOCS1, SOCS2, SREBF2, **TGFB1**, THBS1, TICAM1, TLR2, TLR4, TNFAIP3, TOP2A, UHRF1, UPP1	6.258	2.35E-15
	Immunoglobulin E (IGE)	antibody	**ADAM8**, **ANXA1**, ASB2, BAIAP2, BCAT1, BCL2, BCL3, BIRC5, CAPN2, CCL22, CCL5, CCR5, Cd33, CD80, CD86, CDK19, CDKN1A, CLEC7A, **COL18A1**, CTSK, CX3CL1, CXCL16, DUSP2, DUSP4, EGR1, EGR2, EMILIN1, ENO2, ERRFI1, F2R, FYN, GADD45B, GDF15, HAVCR2, HBEGF, HIP1R, HIVEP3, IL7R, **ITGA5**, **ITGAV**, **ITGAX**, JAK2, JUNB, LRRC38, MDFIC, NCF4, NFKB2, NFKBIE, PDGFB, PILRA, Plpp1, PTGS2, PTPN6, PXMP2, RAI14, RASGRP1, RELB, **RHOD**, RNF180, RUNX1, SERPINB1, SERPINB6, Serpinb6b, SLC11A1, SLC37A2, SOCS1, SPHK1, SRGAP3, STAT5A, **TGFB1**, TLR2, TNC, TNFRSF10A, TNFRSF11B, TNFRSF12A, TNFRSF13B, Tnfrsf22/Tnfrsf23, TRAF1, **TUBB6**, UGCG, **VIM**	6.407	1.9E-23
	Transforming Growth Factor Beta 1 (TGFB1)	growth factor	ABI2, ACTC1, **ADAM19**, **ADAMTS12**, **ADAMTS3**, **ADAMTS4**, ADI1, ADK, ALDH18A1, ALOX12, AMD1, ANGPTL4, **ANKRD1**, ANPEP, **ANXA2**, **ANXA8/ANXA8L1**, ASS1, ATXN1, B3GALT2, BBC3, BCL2, BCL3, BDH1, BGN, BIRC5, BMP6, BUB1,	6.738	6.92E-35

Predicted Activation State	Upstream Regulator	Molecule Type	Target Genes	Activation z-score	p-value of overlap
			BUB1B, C1QA, C3AR1, C5, Calm1, CASP3, CASP4, CBR3, CCL5, CCNA2, CCNB1, CCNB2, CCNE1, CCR5, CCRL2, CD14, CD4, CD44, CD68, CD72, CD80, CD83, CD86, CDH11, CDH19, CDH4, CDK1, CDK5R1, CDKN1A, CDKN2A, CDT1, CELSR2, CENPE, CENPF, CIITA, CKS1B, CLIC4, CLU, **COL18A1**, **COL3A1**, **COL4A1**, **COL4A2**, **COL5A1**, **COL6A1**, **COL6A2**, **COL6A3**, **COL8A1**, COTL1, CPXM1, CSPG4, **CTSB**, **CTSK**, **CTSS**, CTTN, CX3CL1, CX3CR1, CXCL10, CXCR6, CYBB, DAPK1, DBP, DKK3, DUSP4, EDNRA, EEF1A1, EGLN1, EGR1, EGR2, EGR3, EIF4EBP1, ELF3, EMILIN1, ENO2, ESPL1, F2R, F2RL1, FAM110B, FASN, FETUB, FGFBP1, FHL1, FNDC5, FOS, FOSB, FYN, FZD1, GADD45A, GADD45B, GCNT1, GDF15, GEM, GPRC5B, GSDME, GSTA5, HBEGF, HDAC9, HEXA, HGF, HK1, HMGA1, HNMT, HSF2BP, HSPA1A/HSPA1B, HSPA5, HSPB1, IER2, IFI30, IFIT3, IGFBP3, IGFBP4, IGHM, IL10RA, IL12A, IL1RN, IL2RB, IRAK2, **ITGA5**, **ITGAV**, **ITGAX**, **ITGB2**, ITIH5, JUNB, KCNG1, KDELR3, KLF10, KLF15, KLK3, KRT18, KRT7, KRT8, **LAMC2**, LDLR, **LGALS3**, LIMS1, LITAF, LOC102724788/PRODH, **LOXL1**, **LOXL2**, LPL, MAOA, MBOAT2, ME2, MFAP2, MGAT5, MKI67, **MMP12**, **MMP14**, **MMP2**, **MMP3**, MSMO1, MSN, MSTN, **MYL3**, MYOG, NAB2, NCAM1, NCF1, NDRG4, NEGR1, NPAS2, PAPPA, PARP3, PDGFB, PDPN, PILRA, PLAT, PLAUR, PLK2, PLXNC1, PMM1, **POSTN**, PPT1, PRC1, PROM1, PSAT1, PTGER2, PTGS2, PTK2B, PTP4A3, PTPN6, RAB31, RASGRP1, **RHOC**, **RHOD**, RIN1, RND1, RNH1, RRAD, RUNX1, RUNX2, RUNX3, S100A10, S100A4, S1PR2, SAR1B, SBNO2, SELENBP1, SELPLG, SERPINA3, SERPINB1, SHMT1, SLC16A9, SLC1A2, SLC2A1, SLC2A3, SLC7A1, SOCS1, SOX4, SOX9, SPHK1, SSTR2, STAT5A, STAT5B, TAB2, TFAP4, **TGFB1**, TGIF1, THBS1, **TIMP1**, TLR2, TLR4, TMIGD1, TNC, TNFAIP3, TNFRSF10A, TNFRSF11B, TNFRSF12A, **TNNT2**, TOP2A, TP73, TRAF1, TRIM9, **TUBA1A**, **TUBB2A**, UCK2, ULK1, USH1C, VAT1, VCAN, **VIM**, WISP1, WNT11, WNT4, ZFP36L2, ZFPM2, ZNF365		

Predicted Activation State	Upstream Regulator	Molecule Type	Target Genes	Activation z-score	p-value of overlap
	Tumor necrosis factor (TNF)	cytokine	A4GALT, ABR, ACADM, **ADAM8**, **ADAMTS4**, **ADAMTS7**, **ADAMTS8**, AGT, AKR1B10, AMPD3, ANGPTL4, ANPEP, **ANXA1**, ARC, ARHGAP22, ARL6IP5, ASS1, ATF3, B4GALNT1, BBC3, BCKDHA, BCKDHB, BCL2, BCL3, BGN, BID, BIRC3, BIRC5, BMPER, BPGM, BTG2, BUB1B, C3AR1, C5, CA2, CASP3, CASP4, CBR3, CCL22, CCL28, CCL5, CCNE1, CCR5, CD14, CD4, CD44, CD80, CD82, CD83, CD86, CDH11, CDK5R1, CDKN1A, CDKN2A, CERS6, CHRND, CHSY3, CIB2, CIITA, CLEC11A, CLIC4, CLU, CNR2, **COL15A1**, **COL27A1**, **COL3A1**, COLQ, COTL1, CRYAB, **CTSB**, **CTSK**, **CTSS**, **CTSZ**, CTTN, CX3CL1, CX3CR1, CXCL10, CXCL16, CYBB, CYP27A1, CYTIP, DBT, DLL4, **DMD**, DUSP2, DUSP4, DUSP5, EFHD2, EGFR, EGLN1, EGR1, EGR2, EGR3, ELF3, EMP1, EMP2, ERBB2, EXOC3L4, F2RL1, FADD, FASN, FOS, FOSB, FOSL1, FOXO4, FST, FUT4, FYN, GABRA1, GABRG2, GADD45A, GADD45B, GBP6, GDF15, GEM, GPD1, GPD2, GPRC5B, GPX1, GRN, H19, HBEGF, HDAC9, HEXA, HGF, HID1, HLA-A, HLA-E, HPCA, HPGDS, HSPA1A/HSPA1B, HSPA5, IER2, IFIT3, IGFBP3, IGFBP4, IL10RA, IL12A, IL1RN, IL21R, IL7R, IRAK2, IRS2, **ITGA5**, **ITGAV**, **ITGAX**, **ITGB2**, JUNB, KIF20A, KLF10, KLF5, KLK3, KRT18, KRT8, **LAMC2**, LDLR, **LGALS3**, LITAF, LPL, LY96, MAP2K6, MAP3K14, MEOX1, MFHAS1, **MMP12**, **MMP14**, **MMP16**, **MMP2**, **MMP3**, MSLN, MSTN, Mt1, Mt2, MVP, MYOG, NCAM1, NCF1, NFKB2, NFKBIE, NOD2, NQO1, OASL, P2RX5, PAPPA, PC, PCDH7, PDGFB, PDIA4, PDK3, PDPN, PER2, PHLDA1, PLA2G16, PLA2G5, PLAT, PLAUR, PLD3, PLK2, PLXNB2, **POSTN**, PTGS2, PXMP2, PYCARD, RASSF7, RELB, RGS1, RGS2, RND1, RRAD, RRM2, RUNX2, SCUBE2, SELPLG, SERPINA3, SERPINB1, SERPINB8, SGPL1, SLC11A1, SLC15A3, SLC16A2, SLC1A2, SLC22A4, SLC2A1, SLC2A4, SLC40A1, SLC7A1, SLC7A2, SNN, SOAT1, SOCS1, SOCS2, SOX4, SOX9, SPHK1, SQLE, STAT5A, TALDO1, TBXAS1, TFPI2, TGFB1, TGIF1, Tgtp1/Tgtp2, THBS1, TICAM1, **TIMP1**, TLR2, TLR4, TMEM176B, TNC, TNFAIP3, TNFRSF10A, TNFRSF11B, TNIP1, TP63,	7.785	6.19E-32

Predicted Activation State	Upstream Regulator	Molecule Type	Target Genes	Activation z-score	p-value of overlap
			TRAF1, TREM2, TST, TUB, TYK2, UACA, UCP2, UGCG, USP2, **VIM**, WISP1, ZNF365, ZNF750		

Supplementary Table 6. Ingenuity Pathway Analysis – RNAsequencing *mdx* vs *mdx* ^{TG}

*Target genes in red are ECM, ECM-associated, cytoskeletal, or cytoskeletal-associated genes.

Predicted Activation State	Upstream Regulator	Molecule Type	Target Genes	Activation z-score	p-value of overlap
Inhibited	Prostaglandin E Receptor 4 (PTGER4)	G-protein coupled receptor	Ccl7, CXCL10, CXCR4, EGR1, GDNF, GLIS3, HIVEP3, IGF2BP2, PDGFB, **SPP1**	-2.345	0.00765
	Dachshund Family Transcription Factor 1 (DACH1)	transcription regulator	CDKN1A, EGR1, IER2, IGFBP3, TNFAIP3	-2.236	0.00329
	Scaffold Attachment Factor B (SAFB)	Nuclear matrix	BBC3, CNTNAP2, CX3CL1, CXCL10, HEXA	-2.219	0.0146
	Prostaglandin E Receptor 2 (PTGER2)	G-protein coupled receptor	AURKA, CENPE, CLEC4D, CXCR4, EGR1, ESD, **ITGAL**, MELK, **SPP1**	-2.138	0.00207
	SP110 Nuclear Body Protein (SP110)	transcription regulator	CLU, CXCL10, EGR3, MAOA, PANX1, PERP, SOX4, SQSTM1	-2.121	0.0212
Activated	Protein Kinase A (Pka)	complex	ARC, CDKN1A, CXCL10, CYP51A1, DUSP4, EGR1, JUNB, SLC1A2, SOX9	2.975	0.0145
	Transforming Growth Factor Beta 1 (TGFB1)	growth factor	ABCG1, ABI2, ALOX12, AMD1, ASS1, BBC3, C5, CBR3, Ccl7, CD300A, CD68, CDKN1A, CENPE, CLU, CSPG4, CTTN, CX3CL1, CXADR, CXCL10, CXCR4, CXCR6, DEPTOR, DUSP4, EDNRA, EGF, EGR1, EGR2, EGR3, EIF4EBP1, ELF3, ENO2, F2RL1, FABP5, FETUB, FGFBP1, FNDC5, GAL, GDF15, GDNF, GSDME, GSTA5, HEXA, HK1, HMGA1, HSF2BP, HSPA1A/HSPA1BID4, IER2, IER3, IGF2, IGFBP3, IGFBP5, IGHM, IL12A, IRAK2, **ITGAL**, JUNB, KRT7, **LGALS3**, LOC102724788/PRODH, MAOA, Masp1, ME2, MEFV, **MMP12**, MSTN, NAB2, NCAM1, NDRG4, NEGR1, PDGFB, PLA1A, PLK2, PMM1, **RHOC**, RIN1, RNH1, S100A10, S1PR2, SLC16A9, SLC1A2, SLC2A1, SLC2A3, SOX4, SOX9, SPP1, SSTR2, TGIF1, TLR2, TMIGD1, TNFAIP3, TNFRSF12A, TP73, TRIM9, USH1C, VAT1, VCAM1, WNT4, WNT5A	3.085	4.71E-12
	KRAS proto-oncogene (KRAS)	GTPase	CDKN1A, CLU, CRYAB, CX3CL1, CXADR, CXCL10, DUSP4, DUSP5, EGR1, EGR2, F2RL1, FKBP11, FOSL1, GLUL, HEXA, HMGA1, IER3, IGF2, IGF2BP2, JUNB, KCNN4, **LGALS3**, **LMNB1**, LRP8, LYZ, MSLN, NCAM1, NETO2, NFKB2, NGEF, NQO1, PLA1A,	3.101	5.18E-09

Predicted Activation State	Upstream Regulator	Molecule Type	Target Genes	Activation z-score	p-value of overlap
			**RHOC**, RNH1, SOX9, **SPP1**, SQSTM1, STOM, TNFRSF12A, UPP1, ZFAND2A		
	palmitic acid	chemical - endogenous mammalian	BBC3, CD68, CDKN1A, CPE, CXCL10, CYP4F12, ELOVL6, F2RL1, GDF15, IRAK2, SQSTM1, TLR2, TRIM63, UCHL1, VCAM1	3.132	0.000965
	Uncoupling Protein 1 (UCP1)	transporter	AMD1, Cd24a, CD68, CPNE2, CXCL10, EIF4EBP1, ENHO, GDF15, MSS51, ODF3L2, PGD, REEP6, SMOX, SMYD2, STAB2, TPPP, USH1C	3.85	2.11E-06
